# Novel Outcome Measures for Clinical Trials in Cystic Fibrosis

**DOI:** 10.1002/ppul.23146

**Published:** 2014-12-30

**Authors:** Harm AWM Tiddens, Michael Puderbach, Jose G Venegas, Felix Ratjen, Scott H Donaldson, Stephanie D Davis, Steven M Rowe, Scott D Sagel, Mark Higgins, David A Waltz

**Affiliations:** 1 Department of Pediatric Pulmonology and Allergology, Department of Radiology, Erasmus University Medical Center-Sophia Children's HospitalRotterdam, The Netherlands; 2 Department for Diagnostic and Interventional Radiology, Hufeland KlinikumBad Langensalza, Germany; 3 Department of Anesthesia, Critical Care and Pain Medicine, Massachusetts General HospitalBoston, Massachusetts; 4 Department of Pediatrics, Division of Respiratory Medicine, Hospital for Sick Children, University of TorontoToronto, Ontario; 5 Department of Medicine, University of North CarolinaChapel Hill, North Carolina; 6 Department of Pediatrics, James Whitcomb Riley Hospital for Children, Indiana University School of MedicineIndianapolis, Indiana; 7 Department of Medicine, University of Alabama at BirminghamBirmingham, Alabama; 8 Department of Pediatrics, Children's Hospital Colorado, University of Colorado School of MedicineDenver, Colorado; 9 Vertex Pharmaceuticals EuropeAbingdon, UK; 10 Vertex PharmaceuticalsBoston, Massachusetts

**Keywords:** cystic fibrosis, endpoints, outcome measures, imaging, sputum biomarkers, CFTR activity

## Abstract

Cystic fibrosis (CF) is a common inherited condition caused by mutations in the gene encoding the CF transmembrane regulator protein. With increased understanding of the molecular mechanisms underlying CF and the development of new therapies there comes the need to develop new outcome measures to assess the disease, its progression and response to treatment. As there are limitations to the current endpoints accepted for regulatory purposes, a workshop to discuss novel endpoints for clinical trials in CF was held in Anaheim, California in November 2011. The pros and cons of novel outcome measures with potential utility for evaluation of novel treatments in CF were critically evaluated. The highlights of the 2011 workshop and subsequent advances in technologies and techniques that could be used to inform the development of clinical trial endpoints are summarized in this review. Pediatr Pulmonol. © 2014 The Authors. *Pediatric Pulmonology* published by Wiley Periodicals, Inc.

## Introduction

**Figure 1:**
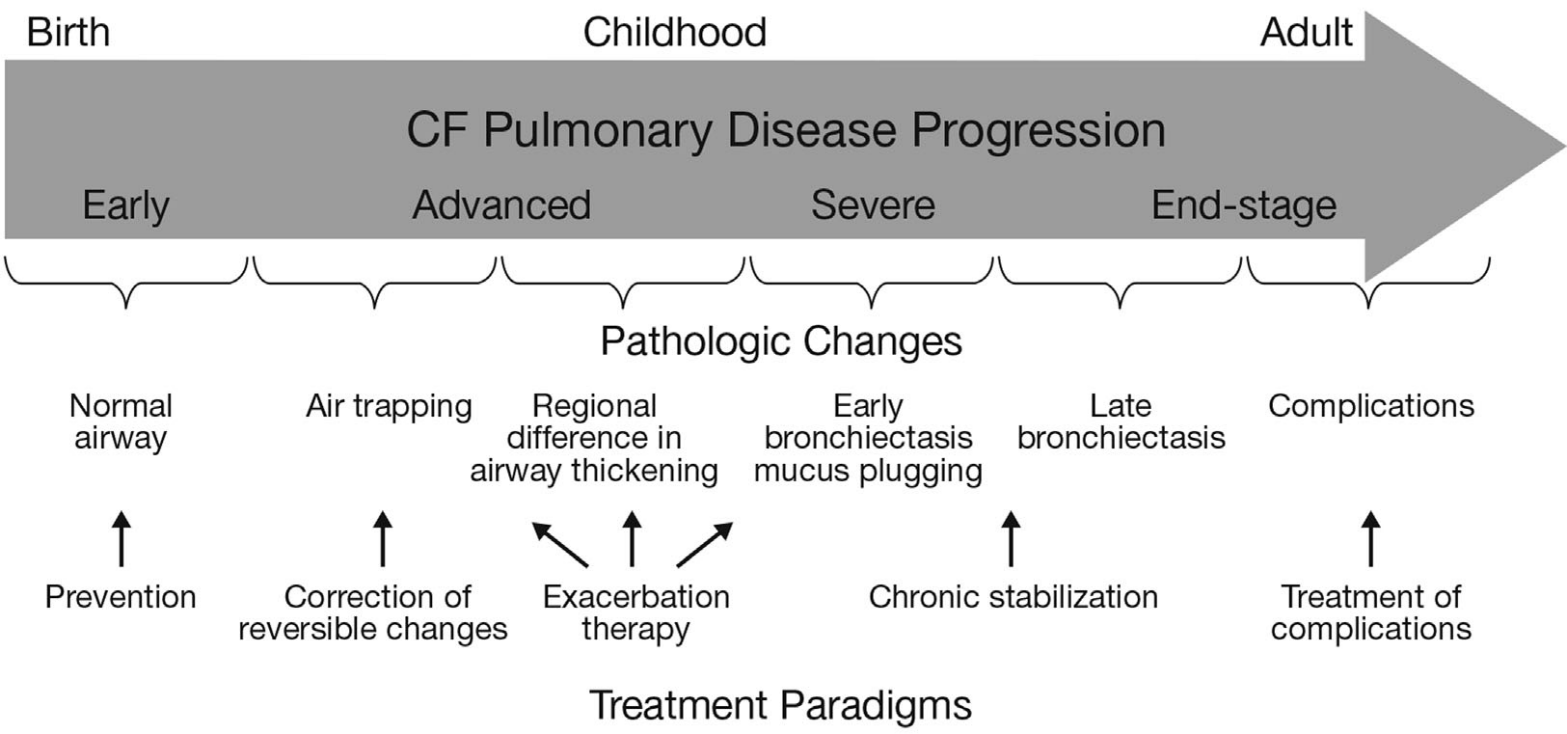
Stages of disease progression and pathologic changes that occur in the airways of patients with CF as they age, along with possible treatment approaches. Reprinted with permission of the American Thoracic Society. Copyright © 2014 American Thoracic Society. Ramsey BW. 2007. Use of lung imaging studies as outcome measures for development of new therapies in cystic fibrosis. Proc Am Thorac Soc;4(4):359–63. Official Journal of the American Thoracic Society.^2^

Cystic fibrosis (CF) is caused by mutations in the gene encoding the CF transmembrane conductance regulator (CFTR) protein, an ion channel that transports chloride ions across epithelial cell membranes. Therapeutic progress has been realized over the last 20 years with improved health, health-related quality of life (HRQoL) and overall survival.^1^ These improvements may, in part, be due to changes in therapeutic approach and patient management. There has been a recent shift in the treatment paradigm, with a reactive approach based on responding to acute declines in respiratory health giving way to a proactive approach of preventing exacerbations and loss of lung function (Fig. 1),^2^ as well as improving the functional and emotional well-being of individuals.^3^

As life expectancy improves and decline in lung function is reduced, the traditional endpoints for studies in patients with CF, such as spirometry parameters, are becoming less appropriate for assessing drug effects.^4^ In addition, newborn screening has been widely adopted and the introduction of disease-modifying therapies aimed at correcting the function of the defective CFTR protein^5^ that could be started early in life seems imminent, particularly following the recent approval of the first CFTR potentiator.^6^ Hence there is a need for novel endpoints that allow detection of clinical benefits starting in young children and continuing into adulthood, which are also acceptable to regulatory authorities. More sensitive outcome measures may assist identification of individuals who could benefit from a novel therapy, reduce the sample size and shorten the duration of intervention studies.^4^

A workshop to discuss novel endpoints for clinical trials in CF was held in Anaheim, California, in November 2011. The pros and cons of novel outcome measures with potential to be used for evaluation of novel treatments in CF were critically assessed. The aim of this review is to summarize the highlights of this workshop, together with more recent developments of clinical trial endpoints for CF, in order to identify potential alternatives that may be useful in future studies.

### Pulmonary Function Testing

Parameters derived from spirometry, such as forced expiratory volume in 1 sec (FEV_1_), still play an important role as outcome measures in clinical trials. However, with improved therapeutic options and earlier interventions, FEV_1_ has become less useful as a tool for differentiating between interventions, as differences in FEV_1_ are reduced, so establishing statistical significance is more difficult. Recently, end expiratory flows such as FEF_25–75_ and FEF_75_ have gained new interest as markers of early lung disease. End expiratory flows show more variability compared to FEV_1_, but they are substantially impaired in early disease and have been shown to respond to therapy in clinical studies.^7^–^9^

Unfortunately, standard spirometry cannot be routinely used in children below the age of 6. For these young children, preschool and infant pulmonary function tests have been developed.^10^

#### Infant Pulmonary Function Testing

The raised volume rapid thoracoabdominal compression (RVRTC) technique (Table1) is a reproducible, repeatable and safe infant pulmonary function test (iPFT), which allows the use of functional outcome parameters for the diagnosis of airflow limitation and monitoring of CF in sedated infants.^11^,^12^ Furthermore, standardized guidelines for measurement and interpretation of results obtained using RVRTC have been published and commercial equipment is now available.^12^

**Table 1:** Techniques Under Investigation For Clinical Studies in Young Children, and the Status of Their Validation

Assessment	Age group	Disease severity (structural or functional changes assessed)	Type of evidence	MCID defined	Test frequency	Safety aspects	Status of standardization (reproducibility, validity)	Cost of equipment
Infant pulmonary function testing (RVRTC)^11^–^18^	<3 years of age	Detection of early functional changes	Well validated	No	Unrestricted	Oral sedation	Well defined	Moderate to high
MBW/LCI^4^,^19^–^35^	All	All	Limited	No	Unrestricted	Oral sedation (optional for young children)	Required for each trial	Moderate to high
CT scans^25^,^36^,^45^–^58^,^60^–^63^	For all ages	Early and advanced structural changes	Well validated	Yes/No	Restricted	Radiation	Well defined and required for each trial	Moderate
MRI^40^–^42^,^57^,^66^–^78^	All (depending on local expertise)	All (morphology and function)	Moderately well validated	No	Unrestricted	Only when contrast media injection is used	Required for each trial	High
PET scan^43^,^44^,^79^–^86^	For children ≥6 years of age	Detection of structural changes as for CT, in addition detection of active inflammation	Few studies in CF available	No	Restricted	Radiation	In development	High
Mucociliary clearance^87^–^93^,^95^	>6 years of age	Early or advanced	Not validated	No	Restricted (4–6 scans per year)	Radiation	Standardized between few sites	Moderate
NPD^119^–^134^	All, specialized at young age	Not applicable	Well validated	No	Unrestricted	Minimal	Well established	Moderate, requires expertise
ICM^135^–^142^	All	Not applicable	Limited	No	Restricted	Requires rectal biopsy	Reasonably standardized	Moderate, requires expertise
Sweat chloride^5^,^120^–^122^,^143^–^149^	All	Not applicable	Well validated	No	Unrestricted	None	Well established	Low

CF, cystic fibrosis; CT, computed tomography; FDA, Food and Drug Administration; ICM, intestinal current measurement; LCI, lung clearance index; MBW, multiple breath washout; MCID, minimal clinically important difference; MRI, magenetic resonance imaging; NPD, nasal potential difference; PET, positron emission tomography; RVRTC, raised volume rapid thoracoabdominal compression.

Through the use of the RVRTC technique, investigators have shown that lung function parameters are significantly diminished in infants with CF, even in those as young as 3 months old.^11^,^13^,^14^ This finding suggests that RVRTC parameters can identify early disease and may therefore serve as useful clinical trial endpoints in infants with CF. Early identification of disease is particularly relevant in CF as therapeutic interventions aimed at preserving lung function may be most effective when administered within the first 6 months of life.^15^ In addition, RVRTC parameters have been shown to be sensitive to interventions in infants with CF. A study has recently revealed that forced expiratory flows and volumes improved following antibiotic therapy for a pulmonary exacerbation in a small subset of infants.^16^ In another recent clinical trial, RVRTC parameters were evaluated as an exploratory endpoint in a subset of patients randomized to either 7% hypertonic saline or isotonic saline for 1 year.^17^ When adjusted for baseline infant lung function, age, gender, height, and weight, the mean change in FEV in 0.5 sec (FEV_0.5_) over 1 year was significantly greater (by 38 ml) in the group treated with 7% hypertonic saline.^17^ However, it should be noted that acceptable measurements were only obtained in 45 of 73 infants in this study, and the clinical significance of a 38 ml difference is not known. Nevertheless, given these recent findings, the commercial availability of equipment, and the promising potential of iPFTs noted in the literature to date, it seems worth pursuing the inclusion of functional parameters as an outcome measure for clinical trials in infants.

However, disadvantages of the RVRTC technique that form a hurdle for use as a primary endpoint must also be considered. Infants must be sedated, equipment is expensive, extensive training of personnel is required, performing measurements is time consuming, and normative data are limited.^11^,^12^,^18^ Despite these challenges, Davis et al. were able to conduct a prospective, longitudinal, observational study with a commercial device in 10 centers in the US.^11^ Personnel at each site underwent rigorous training, and quality control and independent reading of all data were performed by an expert panel.^11^ In this study, key RVRTC parameters were, on average, abnormal in infants with CF compared with healthy historical control subjects.^11^ The authors concluded that infant RVRTC parameters were not yet ready to be adopted as primary efficacy endpoints for multicenter clinical trials, particularly at inexperienced sites and based on acceptability rates.^11^ However, the use of iPFTs as a secondary endpoint should still be strongly considered.

#### Multiple Breath Washout and the Lung Clearance Index

Parameters from the non-invasive, simple multiple breath washout (MBW) tests include functional residual capacity and lung clearance index (LCI) (Table1). LCI is determined by washout of an inert gas during tidal breathing.^19^ As the washout takes longer to complete in the presence of mucus retention or inflammation, LCI increases with disease severity.^19^ LCI measures ventilation inhomogeneity, which helps assess changes in small airways that are not apparent while using spirometry.^20^ LCI has been shown to be superior to spirometry for the detection of early lung disease in CF,^20^–^23^ while MBW has been shown to be equivalent to RVRTC for detecting early disease in infants.^24^

LCI has been demonstrated to correlate with structural lung changes seen on computed tomography (CT) scans in cross sectional studies, suggesting that these techniques have similar sensitivity for the detection of CF lung disease, and that using both methods in individual patients could elicit complementary information.^21^,^25^ A study in pre-school children showed that an abnormal LCI at preschool age could predict lung function abnormalities at school age.^4^ In addition, a study including early school age children demonstrated that LCI correlated with quality of life, and that elevated LCI values could predict pulmonary exacerbations (defined as changes in respiratory status requiring intravenous antibiotics).^26^ Furthermore, LCI may also be a suitable outcome measure to assess early intervention strategies in pediatric patients, as studies among children with CF using normal spirometry have shown LCI to be more sensitive than FEV_1_ for detecting response to treatment with dornase alfa, hypertonic saline, and ivacaftor (in patients with a *G551D-CFTR* mutation and normal FEV_1_).^27^–^29^

One strength of LCI is that it has low variability, both within and between tests,^30^,^31^ indicating that LCI is a suitable endpoint for longitudinal studies.^19^ However, limitations of LCI include that it may require sedation in infants to increase its success rate. Furthermore, LCI is affected by irregular breathing patterns, requirement for expensive equipment such as mass spectrometers with associated software to follow the concentration of the inert gas, and the need for longer washouts as the disease progresses.^19^ While sulfur hexafluoride-based mass spectrometry measurements are considered to be the gold standard,^32^ a number of devices have become commercially available, but will need to undergo thorough validation to assure accuracy of the measurement. Sulfur hexafluoride gas mass spectrometry is not commercially available,^32^ favoring nitrogen-based MBW that only requires 100% oxygen which is readily available in centers. The use of nitrogen-based MBW is increasingly being favored over sulfur hexafluoride-based methods and is more affordable than methods that require mass spectroscopy. A device that determines nitrogen concentration indirectly, by measuring both carbon dioxide and oxygen concentrations, and using Dalton's law of partial pressures, has recently been validated.^33^ In relation to its feasibility, the European CF Society clinical trial network (ECFS-CTN) has selected a commercially available nitrogen MBW system to be used in the network. Over the last year many centers within the network have acquired the system and are currently being trained in its use. Similar efforts are ongoing in North America and training centers have been established in both London and Toronto.

Overall, LCI has potential as a clinical and research outcome measure in young children with CF^4^ and in single-dose, as well as multiple-dose studies.^28^ With regard to the need for sedation, studies that will further assess the suitability of LCI for clinical trials are currently ongoing.^29^,^34^ A recent study demonstrating the utility of LCI as an outcome measure in a multi-center trial.^35^

### Imaging as an Outcome Measure

For the past 40 years, disease progression has been evaluated through lung function tests and plain chest radiographs. The implementation of multi-detector chest CT scan technology has provided clinicians with a more sensitive method for imaging CF lung damage.^36^ Various types of imaging techniques are now used to determine the presence and extent of lung disease in patients with CF, including CT and chest magnetic resonance imaging (MRI). In addition, scoring systems have been developed to quantify and characterize the structural abnormalities detected through CT and MRI in patients with CF at various stages of the disease.^37^–^39^ These systems assess structural changes such as bronchiectasis, trapped air, airway wall thickening, mucus, and opacities. More recently, chest MRI techniques have been developed allowing the assessment of functional characteristics of the lung, as well as the evaluation of lung morphology.^40^–^42^ Positron emission tomography (PET) imaging with [18F]fluorodeoxyglucose ([18]FDG; FDG-PET) can also be used as a non-invasive technique to quantify lung inflammation.^43^,^44^

#### CT Scans

Chest CT scans have been shown to be more sensitive at detecting disease severity than spirometry.^25^,^36^,^45^,^46^ In particular, CT can be used to detect bronchiectasis and trapped air, which reflects abnormal ventilation and perfusion in infants.^47^ Studies have shown that there are weak associations between the presence and extent of structural lung damage and functional parameters.^45^,^47^,^48^ It has also been shown that infection, inflammation and abnormal chest CT findings are already present in a significant proportion of infants with CF at 3 months of age^46^,^49^ and that these early structural changes are progressive.^46^,^50^ These findings suggest that chest CT could be used to detect the presence and extent of structural lung disease, particularly as the majority of infants with lung disease may be asymptomatic.^46^

Evaluation of the ability of chest CT to identify lung abnormalities not detected by spirometry is an important step in validating its use in the diagnosis and monitoring of CF lung disease.^51^,^52^ Two studies showed that chest CT scores are predictive of the respiratory tract exacerbation rate, which is considered a key clinical efficacy outcome measure in CF clinical studies,^51^,^53^ while another showed that the CT scan bronchiectasis score (Brody-II system) was strongly associated with the respiratory tract exacerbation rate.^51^ Moreover, time to first respiratory tract exacerbation and hospitalization was significantly associated with quartiles of bronchiectasis score as recorded by CT scan.^51^

Data from CT scans have also been correlated with survival. In patients screened for lung transplantation, individuals with a higher volume of infection/inflammation-like changes were shown to have a higher risk of dying on the waiting list.^54^ In addition, there was a correlation between HRQoL scores and the presence of structural changes on CT scans.^55^ Recent studies further indicate that CT scans, and identification of bronchiectasis in particular, can be useful in identifying children at risk for worse pulmonary outcomes and could be used to guide treatment decisions.^36^,^56^

The use of chest CTs in clinical trials has been shown to be viable.^55^ However, as with spirometry, use of this technique requires standardization, data transfer, and centralized reading of images. A great advantage of chest CT for clinical trials is that most CF centers have scanners. However, as CT is based on ionizing radiation, the radiation dose has to be minimized, especially in young patients and when scans need to be repeated within a relatively short study period.^36^,^57^ This can be achieved by optimizing low-dose scanning protocols and current CT techniques.^58^ Careful consideration should be given to the balance between radiation exposure and potential benefits.^37^ It is also necessary to define the optimal balance between image modality, image quality, and radiation exposure for each study protocol, at each study site in multicenter clinical trials. In a cohort study of children participating in the Wisconsin CF Neonatal Screening Project, quantitative chest radiography was shown to have excellent sensitivity for detecting abnormal chest CT.^59^ In addition, efforts should be directed at implementing strategies that have been shown to reduce the radiation dose associated with chest CT protocols.^60^–^65^ Using these strategies allows the radiation dose of a chest CT to be reduced to within the range of a routine chest radiograph.^64^,^65^ Regarding its feasibility, over the last year 15 centers within the ECFS-CTN participated in a CT standardization effort. Each center was asked to characterize and optimize the CT scanners using age-specific phantoms; personnel were also trained in the use of a spirometry controlled chest CT protocol. Similar efforts were made in 2 centers in North America and in 9 centers in Australia. A training center has been established in Erasmus MC Rotterdam.

Overall, bronchiectasis and trapped air detected by chest CT are feasible and utilizable surrogate outcome measures for clinical studies of novel treatments for patients with CF (Table1). Furthermore, due to the increased sensitivity of CT scans compared with spirometry, smaller sample sizes will be needed in clinical studies employing CT scan endpoints.^46^ As CT is a sensitive method of detection, it may be useful early in life to detect signs of disease and act as a trigger for initiating therapy before lung function measures, such as FEV_1_, become noticeably impaired.

#### MRI

Pulmonary MRI has been introduced as a research and diagnostic tool, primarily to overcome the limitations of CT scans.^41^ However, pulmonary MRI also has some limitations, such as the signal diminishing deeper into the lung, respiratory and/or cardiac motion artifacts, and magnetic field distortions due to lung parenchyma.^41^ Over the last decade, technical advances have addressed these limitations in functional and morphologic assessment of various pulmonary diseases, including airway diseases.^41^ In particular, motion artifacts can be reduced by faster imaging, so that controlled breathing is no longer necessary for acceptable images.^66^

In patients with CF, MRI is useful for detecting morphologic changes in airways and lung parenchyma, in particular inflammation and mucus plugging,^67^ and can be used in patients of almost all ages.^42^,^68^ In cross-sectional studies, there is a strong correlation between MRI and CT results in patients with CF.^42^ Both MRI and CT are able to detect most large morphologic changes in the CF lung, but MRI is less sensitive for the detection of small airway disease.^57^,^69^–^71^ New sequences are in development that might further improve the resolution of MRI.

MRI has no radiation exposure, a clinically acceptable scan time (15–30 min), and is superior to CT for the assessment of functional changes such as altered pulmonary perfusion.^57^ Further, a reproducible morpho-functional MR-scoring-system has been developed, allowing CF lung disease over a broad severity range to be monitored,^40^,^68^ although hyperperfusion is not taken into account.

MRI is also sensitive to the effects of treatments including antibiotics.^72^ The above mentioned features of MRI may make it particularly suitable for monitoring the course of functional pulmonary changes, such as pulmonary perfusion in response to investigational therapeutic interventions, and assessing other treatment effects in clinical trials. A recent study in infants and pre-school children with CF demonstrated that MRI was able to detect abnormalities in lung structure and perfusion, as well as response to treatment for exacerbations.^68^ These findings confirm the potential of MRI for non-invasive monitoring and as an outcome measure in interventional trials for early CF lung disease.^68^

Overall, although MRI is a feasible and utilizable surrogate outcome measure for clinical studies in patients with CF (Table1), further validation and standardization are still needed.^73^ In addition, while there are no age limitations to the use of MRI, imaging of children below the age of 6 years remains challenging. New methods are in development, but are not yet in routine use.^74^ Experimental lung imaging techniques that provide additional information on ventilation beyond conventional MRI, such as hyperpolarized noble gas (^3^He and^129^Xe) MRI^75^–^77^ and Fourier decomposition MRI,^78^ are currently being evaluated.

#### PET Scanning

The utility of the FDG-PET scan is based on its unique ability to image active infection and inflammation in the lungs.^44^,^79^ In particular, neutrophil (the predominant inflammatory cell in the lungs of patients with CF) activation can be detected using FDG-PET.^44^ Indeed, FDG uptake in the damaged lung has been correlated with uptake by neutrophils and may also be a biomarker of eosinophilic inflammation.^79^,^80^ In patients with CF, a decline in FEV_1_ has been associated with the rate of FDG uptake.^81^ In contrast, adult patients with stable CF do not show enhanced FDG uptake compared with control patients, despite high sputum neutrophil levels.^82^

As the combination of CT and PET scans enables the localization of inflammation to anatomical hotspots, the use of hybrid FDG-PET/CT scanning was evaluated to monitor lung inflammation and/or infection in patients with CF.^83^ This study reported the presence of localized areas of increased uptake of FDG that may represent active focal infections or inflammatory processes in the lungs.^83^ Moreover, in this study, the resolution of the acute infection resulted in either a disappearance or great reduction in the high-intensity areas of uptake.^83^ These findings were recently confirmed in a FDG-PET/CT study in 20 pediatric patients treated for a pulmonary exacerbation.^43^ The above evidence, in conjunction with clinical observation, has led to the suggestion that FDG-PET/CT scanning may be more valuable than CT for assessing responses to antibiotic treatment in CF patients with acute lung infection.^83^ Furthermore, FDG-PET/CT imaging could be enhanced by quantitative measurements of regional ventilation and perfusion, to increase sensitivity for detecting functional changes in the lungs, as demonstrated previously for PET scanning.^84^–^86^

However, FDG-PET/CT exposes patients to relatively high doses of ionizing radiation, which limits its repeated use (Table1). The combination of PET and MRI may overcome this limitation as it could provide valuable results with reduced radiation burden, which is of particular importance in children where radiation exposure must be minimized.

The advantages of FDG-PET and FDG-PET/CT scans warrant further research. The development of improved image analysis methods may help to validate this tool as an endpoint for clinical trials.

### Mucociliary Clearance

Although impaired mucociliary clearance (MCC) is a hallmark of CF, current evidence suggests that MCC varies significantly in children with CF lung disease and normal pulmonary function, with MCC values reported both within and below the normal range in this cohort.^87^ Furthermore, published studies on MCC as an outcome measure have used different methods and yielded inconsistent results.^88^–^90^ Robinson et al. demonstrated a global reduction in MCC in CF patients regardless of their lung function, including from the large airways.^90^ In contrast, Donaldson et al. demonstrated that the 1 hr mucus-clearance rate, which is dominated by large-airway clearance, did not differ significantly between CF patients and controls, whereas clearance in peripheral lung regions and cumulative mucus clearance at 24 hr (both reflective of the smaller airways) were significantly reduced.^88^ Finally, another study showed that long-term lung clearance measured over 21 days was not slower in patients with CF than in healthy subjects.^89^ In this study, greater clearance was reported in CF patients on days 1–7 and no difference in clearance was found between groups from day 7 to day 21.^89^ Potential interpretations of the absence of a difference in particle clearance after 7 days include the absence of a small airway MCC problem, or that clearance via mechanisms other than mucociliary transport (e.g., macrophage-mediated clearance) dominate during these very long time domains.^89^

MCC has several challenges that must be addressed before use in clinical trials. Particle delivery and the resulting pattern of lung deposition are critical determinants of the observed clearance rate in MCC studies, and therefore must be carefully controlled.^91^ Particle deposition depends on aerosol characteristics (average particle size, distribution of size), the breathing pattern used during inhalation (flow rate, tidal volume), and anatomical features of the airways (i.e., degree of obstruction and lung size).^91^ Although total deposition tends to be equivalent between patients with CF and controls, scans have shown that deposition is patchy in patients with CF. Particle clearance is also likely to be heterogeneous,^89^,^91^ potentially complicating the characterization and comparison of clearance rates. To minimize intra- and inter-subject variability in particle deposition, aerosol delivery to the bronchial airways must occur in a reproducible manner.^91^ Variation in aerosol delivery methods likely explains differences between prior studies, and failure to control these variables can degrade the ability to accurately characterize the response to therapeutic interventions. A further limitation of MCC as an endpoint is spontaneous cough and associated additional mucus clearance that has been reported as a main adverse event following inhaled medications.^87^ Finally, MCC is not sensitive to all therapeutic agents; while sensitive to agents that effectively change airway surface liquid hydration, it was not altered by dornase alfa in previous studies.^92^,^93^

Despite these limitations, recent findings suggest that MCC has the potential to be used as an outcome measure in CF clinical trials. Results from one study using MCC to assess the efficacy of hypertonic saline suggest that there is an association between improvements in MCC and lung function tests.^88^ In this study, MCC, FEV_1_, and forced vital capacity significantly improved over treatment time in patients receiving hypertonic saline, but not in those who received the ineffective combination of amiloride and hypertonic saline.^88^ In the recent GOAL study, the profound effect of restoring CFTR function on MCC, using ivacaftor in patients with the G551D mutation, also demonstrated the tight link between CFTR function and MCC.^94^ In this multicenter study, MCC more than doubled within 1 month of starting ivacaftor, and this effect was maintained after 3 months of treatment. These data suggest that MCC measurements may provide a useful tool for the study of novel CFTR modulators in the therapeutic pipeline.

At present, measurement of MCC is a promising, but incompletely developed, biomarker for CF clinical research (Table1). A standard operating procedure has recently been developed, which has facilitated the conduct of multicenter studies.^95^ It should also be borne in mind that MCC is not sensitive to all therapeutic agents, and therefore its application must be used selectively in future clinical trials.

### Biomarkers as Outcome Measures

#### Sputum Biomarkers

Sputum is easily obtainable and a rich source of biomarkers of inflammation and infection in patients with CF.^96^–^98^ As airway inflammation plays a central role in CF lung disease, sputum biomarkers of inflammation that can be used to monitor disease activity or evaluate response to therapy would be valuable. Biomarkers may also provide further insight into the pathophysiology of CF lung disease.

Findings from small single-center studies are limited, but support an association between sputum biomarkers and disease status in CF, as determined by pulmonary function tests, chest radiograph scores, HRQoL measures, and illness severity scores (e.g., Shwachman–Kulczycki score).^97^ Significant correlations between FEV_1_ and sputum inflammatory measures, including neutrophil counts, interleukin (IL)-8, and neutrophil elastase, have also been demonstrated in a diverse CF population across multiple centers participating in four CF clinical trials.^99^

Several studies have demonstrated good reproducibility of cell counts and inflammatory mediators in induced sputum.^97,100–102^ Emerging longitudinal analyses of sputum biomarkers provide variability estimates over time, allowing investigators to derive sample size calculations for interventional trials.^103^,^104^

Reductions in sputum biomarkers following therapeutic intervention have been demonstrated in a number of clinical studies.^105^–^107^ Following intravenous antibiotic therapy in patients with CF, reductions in neutrophil counts, IL-8 concentration and neutrophil elastase activity were associated with improvements in FEV_1_.^106^ Importantly, sputum induction was relatively well tolerated in CF patients, even during acute pulmonary exacerbations.^106^ In the initial US azithromycin trial, there were modest differences in sputum elastase between the placebo and treated groups at the end of treatment in favor of the azithromycin group, suggesting that azithromycin may exert an anti-inflammatory effect by preventing a worsening of protease-mediated inflammation over time.^107^ Two other studies, one investigating the anti-inflammatory effects of ibuprofen,^108^ the other examining the CFTR potentiator ivacaftor in G551D subjects,^94^ did not show significant changes in sputum biomarkers of airway inflammation. While these studies raise concerns about the utility of sputum biomarkers, the lack of significant changes could also be attributed to the relatively short treatment periods and the need for more prolonged therapies before substantially impacting airway inflammation. Other potential sputum biomarkers that might be relevant to CF clinical trials include biomarkers of structural injury and matrix degradation;^109^ physical properties, including wet and dry weight, surface tension and impedance; rheology (viscosity, elasticity), and microparticles;^110^ as well as biochemical constituents besides inflammatory mediators (mucins, fibrin, DNA).

Overall, sputum assessments provide a non-invasive endpoint for assessing novel therapies for CF, especially in patients over 10 years of age. Ongoing investigations into the variability and reproducibility of this technique are a practical step before widespread application in clinical trial methodology. In addition, longitudinal analyses are essential for the validation of biomarkers of inflammation, as correlates of disease severity and progression. Data are now emerging from ongoing investigations evaluating the ability of sputum biomarkers to predict key clinical events in CF, including lung function decline,^104^,^111^ pulmonary exacerbations,^103^,^105^ development of bronchiectasis^47^ and even survival.^103^ Taken together, these data provide strong support for the use of sputum biomarkers of airway inflammation as tools to monitor disease activity and as outcome measures in CF clinical trials.

#### Blood-Based Biomarkers

Serum and plasma biomarkers have the potential to provide a relatively non-invasive means of evaluating pulmonary inflammation and infection in patients with CF. Several circulating biomarkers have been investigated, including C-reactive protein, serum amyloid A, calprotectin, neutrophil elastase antiprotease complexes, plasma sCD14, a protein complex containing alfa-1 antitrypsin and CD16b (AAT:CD16b), and cytokines including IL-6 and IL-8.^112^–^115^ A recent systematic review summarized the results of studies that have used blood-based biomarkers to monitor response to treatment during pulmonary exacerbations.^116^ In a clinical trial in CF patients 6–18 years of age uninfected with *Pseudomonas aeruginosa*, azithromycin significantly reduced circulating neutrophil counts and systemic markers of inflammation including C-reactive protein, serum amyloid A, and calprotectin.^117^ Reduction in these inflammatory markers correlated with improvements in lung function and weight gain, providing indirect evidence that these changes were associated with clinically meaningful outcomes. This was the first study to demonstrate the utility of a panel of systemic inflammatory markers in a CF interventional trial and these data provide evidence that systemic biomarkers have added value and should be included in future CF clinical trials.

#### Exhaled Breath Condensate

Exhaled breath condensate (EBC) may provide a potential source of biomarkers that could be useful in a variety of diseases, including CF.^118^ Several potential EBC biomarkers have been investigated in CF, including EBC pH, fractional exhaled nitric oxide, leukotriene-B4, 8-isoprostane, hydrogen peroxide, interferon-γ, IL-10, IL-4, tumor necrosis factor, purines and glucose.^118^ At present there is insufficient evidence to support the use of any of these biomarkers as outcome measures in clinical studies.

### Assessment of CFTR Activity

#### Nasal Potential Difference

It is well established that CF is caused by mutations in the CFTR gene, resulting in disruption of chloride and bicarbonate transport across epithelial cell membranes.^119^ These abnormalities can be evaluated by measuring the transepithelial potential difference (PD) across the nasal epithelium. The degree of CFTR dysfunction, as measured by nasal PD, correlates with the number and severity of CFTR gene mutations.^120^ Measurement of nasal PD has therefore proved useful in clinical studies of novel CFTR therapeutic agents^121^–^124^ and is used in clinical practice as one of the diagnostic criteria for CF.^125^–^127^ However, there are several different methods for measuring nasal PD, as well as important considerations with respect to its use in clinical trials, such as the requirement for well-trained and dedicated personnel to provide accurate assay results.^128^ Commercially-available equipment, which has made this measurement easier to perform, is now in use.^129^ The agar nasal catheter has shown greater reliability than the perfusion nasal catheter for measurement of nasal PD,^130^ and its sensitivity and specificity are generally understood.^131^,^132^ The tool has been particularly useful for evaluating the response, including the effect of different doses of the CFTR potentiator ivacaftor, in G551D CF patients.^121^

PD measurements can also be made in the lower airway via bronchoscopy,^133^,^134^ thus permitting direct assessment of the airways. This could be particularly important for evaluating the effect of inhaled drugs or gene therapy in which only pulmonary delivery is expected. A study has shown that invasive bronchoscopic methods can be utilized safely and reliably in children as young as 1 year of age, albeit under anesthesia.^133^ Therefore, this measure could be a useful functional endpoint assay for studies of either CFTR gene transfer or for future trials evaluating inhaled therapeutics.

#### Intestinal Current Measurement (ICM)

Intestinal current measurement (ICM) was introduced about two decades ago as an ex vivo diagnostic method for CF and has been the subject of renewed interest.^135^–^140^ The technique can distinguish pancreatic-sufficient individuals, indicating its utility in quantifying patient phenotype.^141^ ICM has some advantages over nasal and lower airway PD techniques, including easy access to intestinal tissue in all age groups and minimal tissue damage or remodeling triggered by bacterial or viral infections.^136^ Moreover, ICM allows novel CFTR therapies to be studied in native human epithelium ex vivo without increasing risk to the patient and is able to detect low levels of functionally active CFTR.^136^ The potential use of ICM in clinical trials is being evaluated, and has been combined with protein detection by Western blotting.^136^ In addition, standardized guidelines for this technique are now available, following collaboration between the European CF Society Diagnostic Network Working Group, the European CF Society Clinical Trials Network and the CF Foundation Therapeutics Development Network.^142^

#### Sweat Test

Determination of sweat electrolytes has been performed in clinical laboratories for over 40 years and remains the gold standard diagnostic test for CF.^143^ The measure provides a sensitive indicator of CFTR activity, and correlates well with the CF phenotype.^120^ Sweat chloride measurements are feasible in multicenter clinical trials,^5^,^121^,^122^,^143^ and use of the macroduct collection system allows analysis in a central laboratory, facilitating standardized methodology among centers. Furthermore, the procedure does not place a significant burden on patients and is rarely associated with complications.^143^ Several lines of evidence suggest that sweat chloride is a robust outcome measure in clinical trials evaluating agents directed at restoring CFTR function.^5^,^121^,^122^ As sweat chloride levels change significantly with small changes in CFTR activity, as seen in genotype–phenotype correlations,^120^ their determination allows the evaluation of the effect of CFTR modulators with relatively low activity,^122^ providing a reasonable assay for dose-response relationships. However, change in sweat chloride has not proven to be predictive of lung function response on an individual basis in studies involving a CFTR potentiator.^144^ Recently, sweat rate and sweat conductivity have been determined in conjunction with sweat chloride levels, to assess the secretory function of the sweat gland.^145^,^146^ These techniques may be particularly well suited to detecting altered glandular activity, although this needs to be demonstrated in prospective studies.

In conclusion, application of ion channel measurements in clinical trials appears feasible given its established use, favorable safety and acceptability profile and validity (Table1). Widespread use in recent years has improved understanding of its sensitivity, specificity, and reproducibility.^121^,^122^,^145^,^147^,^148^ However, the need for well-trained personnel and use of accepted standard operating procedures should be employed when including sweat chloride as an outcome measure in clinical trials. In addition, concerns regarding whether pharmacodynamic activity in the absorptive epithelium of the sweat gland correlates with activity in the secretory epithelia of the airway and other organs still need to be addressed.^149^ This could be achieved by characterizing CFTR, and its response to novel therapies, in different tissues. Other questions include whether tissue (i.e., skin) penetration or differences in the absorptive function of CFTR following pharmacologic rescue of F508del may impair its ability to detect the efficacy of multi-agent therapy.

## Conclusion

Spirometry is the standard clinical trial endpoint in older children and adults with CF. However, it is now well recognized that, over time, decline in FEV_1_ has become relatively small, thus making it an insufficiently sensitive marker of CF to serve as a primary endpoint in clinical studies. There is consequently an urgent need for alternative sensitive and accurate surrogate outcome measures that detect early lung disease and track disease progression through early childhood into adulthood.^4^

Ideally endpoints must be accurate, reproducible, sensitive, and reflect patient function and survival. They should also predict the efficacy of therapy,^99^,^145^,^147^ as well as being feasible for use in clinical studies with small patient numbers, non-invasive and inexpensive. The most promising and feasible new sensitive outcome measures that can be used in today's clinical trials to measure severity of CF lung disease in (young) children and adults, are the LCI and the bronchiectasis scores derived from chest CT. Tests to measure CFTR activity, such as the sweat test, are of key importance to establish the effect of CFTR modifiers on CFTR function.
